# Fully automated left ventricle function analysis with self-gated 4D MRI

**DOI:** 10.1186/1532-429X-18-S1-P37

**Published:** 2016-01-27

**Authors:** Yuhua Chen, Jianing Pang, David Neiman, Yibin Xie, Christopher T Nguyen, Zhengwei Zhou, Debiao Li

**Affiliations:** 1 grid.50956.3f0000000121529905Biomedical Imaging Research Institute, Cedars-Sinai Medical Center, Los Angeles, CA USA; 2 grid.25879.310000000419368972Deparment of Computer and Information Science, University of Pennsylvania, Philadelphia, PA USA; 3 grid.19006.3e0000000096326718Bioengineering, University of California, Los Angeles, CA USA; 4 grid.14003.360000000099041312Electrical Engineering, University of Wisconsin, Madison, WI USA

## Background

Left ventricle (LV) function parameters such as stroke volume and ejection fraction (EF) are vital physiological parameters in the management of heart diseases. In cardiac MR, these parameters are currently derived from multiple short-axial cine images acquired under breath hold, which requires extensive scan planning and patient cooperation. The post-processing workflow is also labor-intensive as a human operator is required to manually trace the endocardium border on every slice. Also, the calculated LV volume may be inaccurate due to low resolution in slice direction and slice mismatch from inconsistent breath hold positions. In this work, we propose to combine a recently developed free-breathing 4D MRI technique and atlas-based image segmentation to calculate the LV function parameters fully automatically.

## Methods

MR data were collected with a self-gated, contrast-enhanced, spoiled gradient echo pulse sequence using a 3T clinical scanner (Siemens Healthcare, Erlangen, Germany) on healthy volunteers (N = 5) with informed consent and IRB approval. From each dataset, a 16-phase whole-heart cine series was reconstructed using the methods proposed in [1]. Then, the following steps were taken to automatically derive the LV volume from the 16-phase series: first, an unbiased template was constructed by iteratively averaging all cardiac phases with motion correction; second, the template LV was segmented out using atlas-based joint label fusion [2] with a diffeomorphic registration [3]; finally, the template LV segmentation was warped back to the 16 phases using the inverse transforms and corrective learning [4]. We conducted a leave-one-out cross validation to evaluate the consistency between the automated and expert manual segmentations. The metrics used included Dice coefficient, mean minimum distance (MINDIST), and the agreement of the computed LV volumes.

## Results

**Figure 1:**
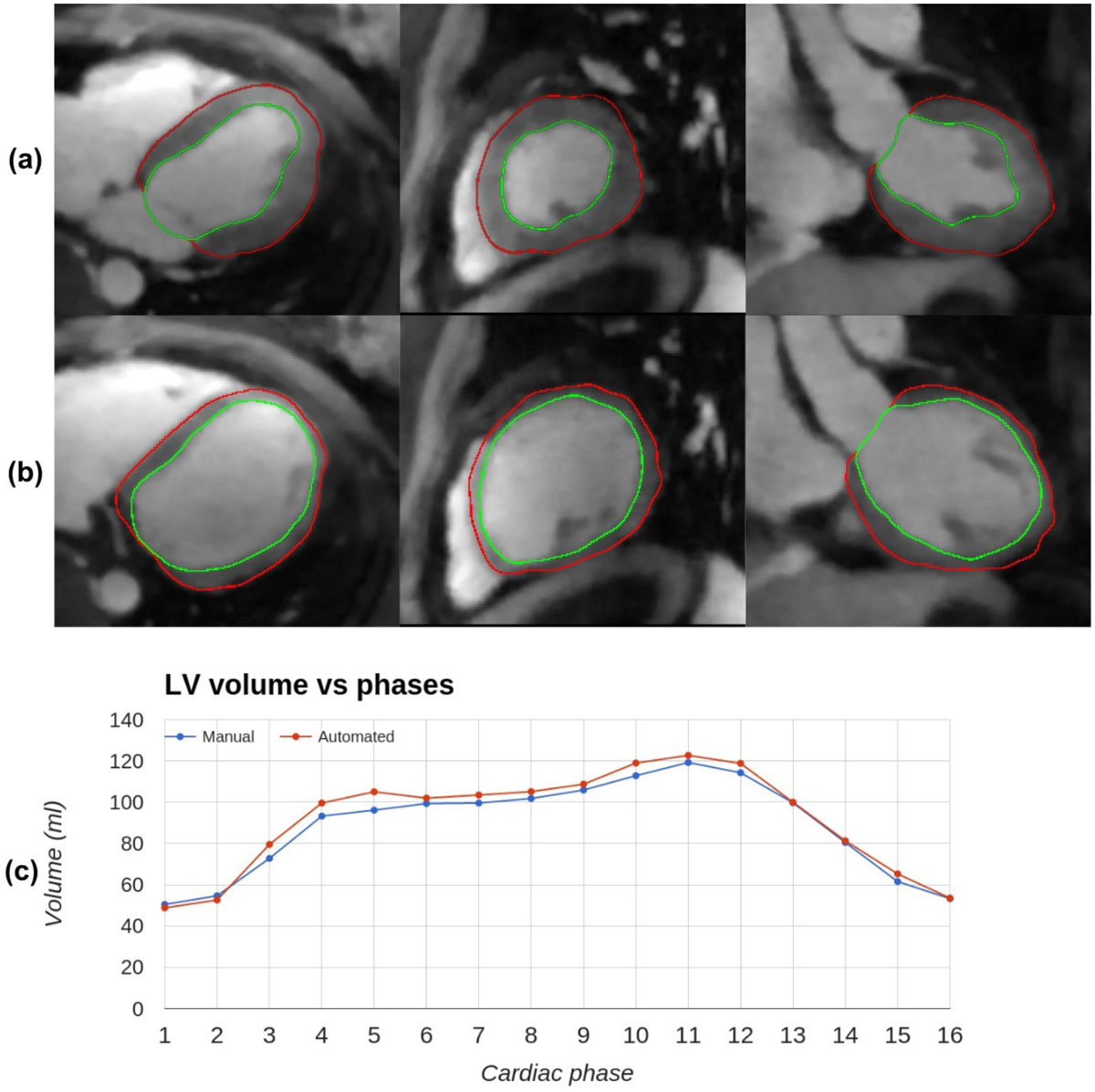
**Automatically segmented systolic (a) and diastolic (b) phases from an example case, shown in axial, sagittal, and coronal planes**. The endocardial and epicardial borders are shown in green and red, respectively. The 16-phase LV volume measurements from manual and automatic segmentations are shown in (c).

**Figure 2:**
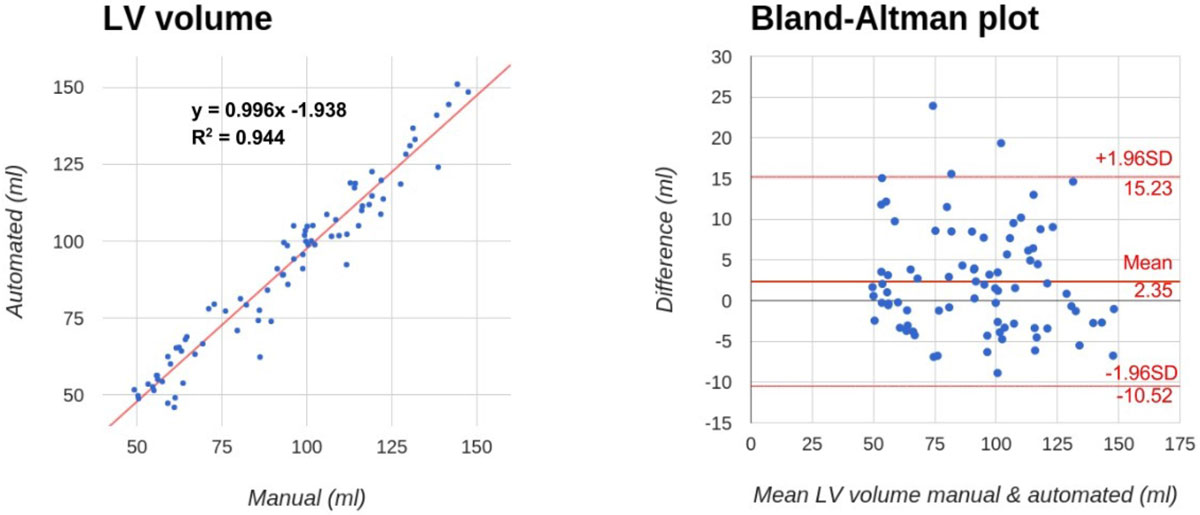
**The manually and automatically measured LV volumes demonstrated excellent correlation and agreement, as shown in the regression analysis (left) and Bland-Altman plot (right)**. No significant difference was found between the two measurements.

Fig. 1 shows an example segmentation result and the calculated LV volumes throughout the entire cardiac cycle. Among all subjects and cardiac phases, the mean Dice coefficient and mean MINDIST were 0.93 ± 0.03 and 0.13 ± 0.08 mm, respectively. As shown in Fig. 2, the LV volumes computed using the proposed method and manual segmentations demonstrated excellent correlation and agreement, with correlation coefficient = 0.97 and mean percent difference = 5.86%. The mean absolute difference in EF measurements from the two groups was 3.88%.

## Conclusions

In this work, we developed a fully automated method to segment LV from 4D MR images. The proposed technique showed excellent agreement with expert manual segmentations in our preliminary evaluation, and may emerge as a favorable method for fast and accurate LV function analysis with minimal operator dependency. Future works will be focused on performance tuning and further validations on both healthy subjects and patients.
